# Scaling in topological properties of brain networks

**DOI:** 10.1038/srep24926

**Published:** 2016-04-26

**Authors:** Soibam Shyamchand Singh, Budhachandra Khundrakpam, Andrew T. Reid, John D. Lewis, Alan C. Evans, Romana Ishrat, B. Indrajit Sharma, R. K. Brojen Singh

**Affiliations:** 1 Centre for Interdisciplinary Research in Basic Sciences, Jamia Millia Islamia, New Delhi-110025, India; 2 School of Computational and Integrative Sciences, Jawaharlal Nehru University, New Delhi-110067, India; 3 McConnell Brain Imaging Center, Montreal Neurological Institute, McGill University, Montreal, QC H3A 2B4, Canada; 4 Institute of Neuroscience and Medicine (INM-1), Research Centre Jülich, Jülich, Germany; 5 Department of Physics, Assam University, Silchar-788011, Assam, India

## Abstract

The organization in brain networks shows highly modular features with weak inter-modular interaction. The topology of the networks involves emergence of modules and sub-modules at different levels of constitution governed by fractal laws that are signatures of self-organization in complex networks. The modular organization, in terms of modular mass, inter-modular, and intra-modular interaction, also obeys fractal nature. The parameters which characterize topological properties of brain networks follow one parameter scaling theory in all levels of network structure, which reveals the self-similar rules governing the network structure. Further, the calculated fractal dimensions of brain networks of different species are found to decrease when one goes from lower to higher level species which implicates the more ordered and self-organized topography at higher level species. The sparsely distributed hubs in brain networks may be most influencing nodes but their absence may not cause network breakdown, and centrality parameters characterizing them also follow one parameter scaling law indicating self-similar roles of these hubs at different levels of organization in brain networks. The *local-community-paradigm* decomposition plot and calculated *local-community-paradigm*-correlation co-efficient of brain networks also shows the evidence for self-organization in these networks.

One of the most important issues in the study of brain networks is the origin of functional modules, organization of these modules, and their functional relationships. Brain networks, constructed from various experimental studies on brains of different species, exhibit hierarchical features (highly modular structure)^1^,^2^, and these modules are believed to be sufficiently isolated to enable them to perform independent functions^3^. These sparsely distributed modules in brain network are shown to exhibit small-world topology, which have large local clustering co-efficients and very small path lengths^4^,^5^, and it may allow the modules to perform independent functions^6^. On the other hand, studies on brain networks derived from functional magnetic resonance imaging (fMRI)^7^,^8^,^9^, structural MRI^10^, and diffusion tensor MRI^11^ show small worldness in brain networks which seems inconsistent with the observed high modularity. High clustering in small worldness, which is a local parameter, could not explain the global high modularity of brain networks^1^,^12^,^13^; and short path length in small worldness is also not suitable for strong modularity^13^,^14^. Since the strong modularity corresponds to *large world*, the hierarchically organized, highly clustered, nearly isolated, and self-similar set of modules are shown to be weakly tied among themselves^14^, as a consequence of which the network preserves the small-world properties^13^. Therefore, the weak ties among the modules are believed to maximize the information transfer among the modules with minimum wiring cost, and also allow to maintain small-world topological characteristics^13^,^15^. Further, these weak connections among modules in brain networks compel limited propagation of avalanche of neurons among the modules and are modular size dependent^16^.

Fractality or self-similar structures in a complex network could be one property which can explain functional relationships of a larger network down to the fundamental structure through different levels of organization^14^,^17^. Scaling and renormalization theory can probably highlight the importance of information flow in a complex network and its self-organization^18^. It has been shown that hierarchical organization of modules in functional brain networks (fMRI) show fractal properties^13^. This fractal organization of modules in the network keeps hubs tightly bound inside corresponding modules, and use low-degree nodes as inter-modular connectors showing *disassortative topology*^19^. Further, it has been shown that networks (forty seven different networks) which are structurally self-organized follow fractal law in parameter space of network size and edge density^20^. However, whether organization of modules and sub-modules at different topological levels follow fractal nature or not is still an open question. Further, whether the fractal properties exist in brain networks of different organisms (lower to higher level organisms) is not fully investigated.

In this study, brain networks of four different species (from lower to higher level of brain organization), namely, *C. elegans*, cat, macaque monkey, and human were investigated. Scaling laws and fractal rules were applied on several topological parameters to investigate the self-organization and fractal properties of the brain networks. Recently, Cannistraci *et al*.^21^ proposed a technique known as *local-community-paradigm* (LCP) to estimate the size of local communities and their information, which can be used as an indicator self-organization in complex networks. We implement this technique to brain networks, and we study the robustness of self-organization not only on complete networks but also at various levels of network organization.

## Results

**Figure 1:**
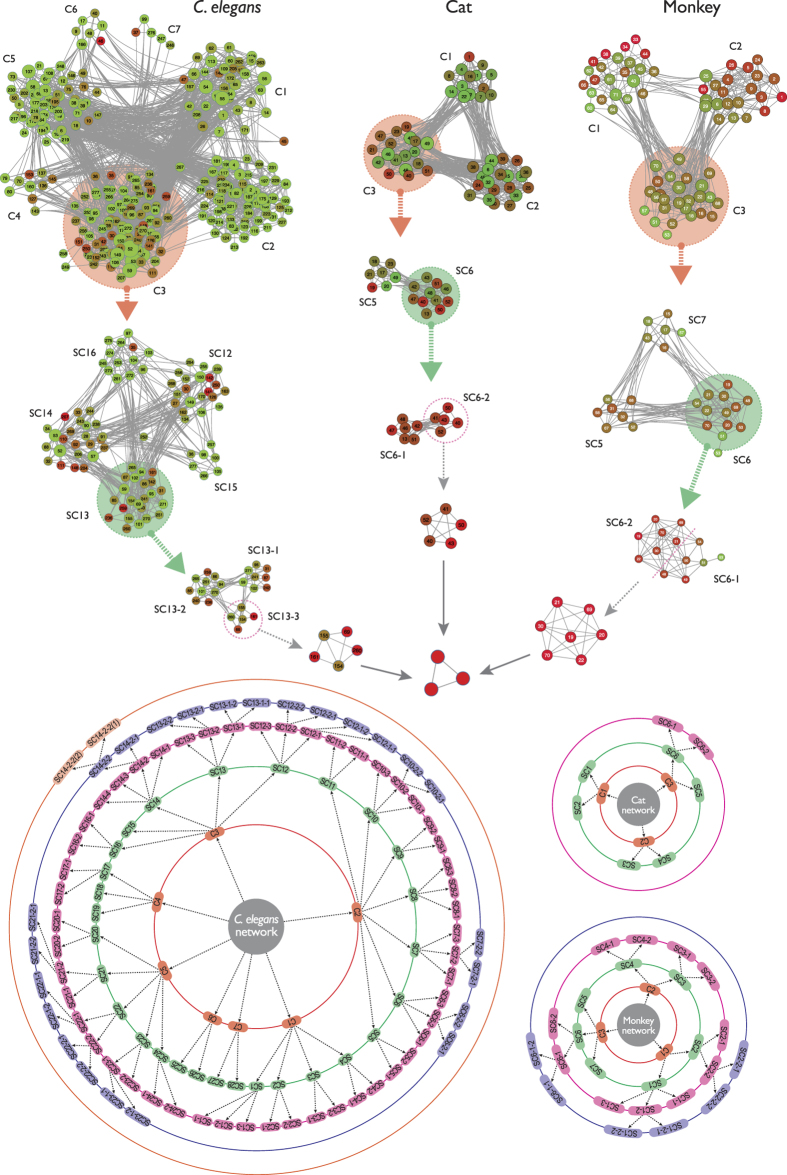
Hierarchical organization of brain networks of *C. elegans*, cat, and monkey at different levels. The upper parts show the topological arrangement of modules and sub-modules at various levels of organization (one way of largest module and sub-module) till the motif level. The lower parts show the organization of all modules and sub-modules at different levels (levels are indicated by circles) of the brain networks of the three species.

**Figure 2:**
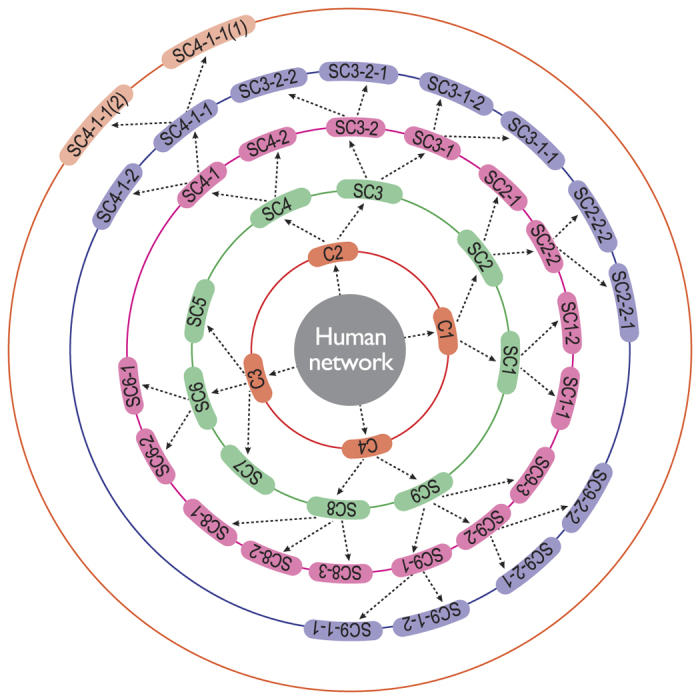
Modules and sub-modules at various levels of organization (with levels indicated by circles) of the brain network of human species.

**Figure 3:**
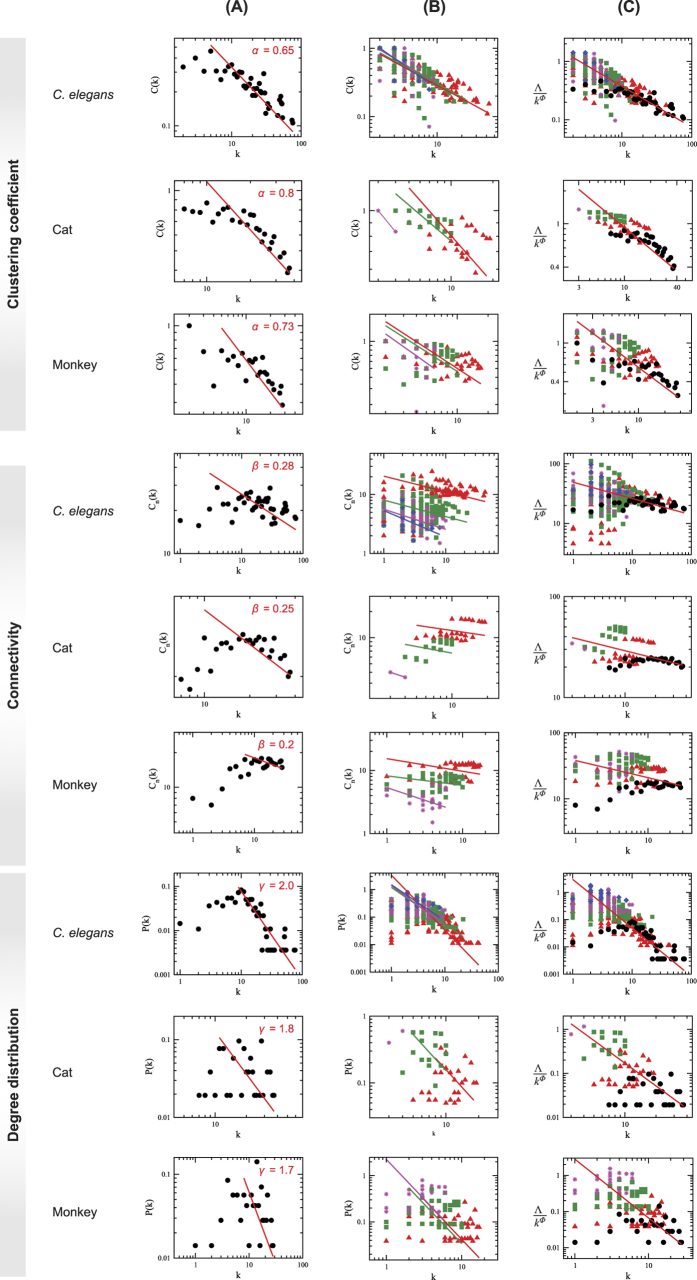
Topological characteristics of brain networks of three species, *C. elegans*, cat, and monkey: (**A**) for whole brain network, (**B**) for modules and sub-modules at various levels of network, (**C**) scaled for all modules and sub-modules in all levels to a single plot. The first three upper rows of panels are for clustering co-efficient, next three rows are for neighborhood connectivity, and the last three rows are for probability of degree distribution of the three species.

**Figure 4:**
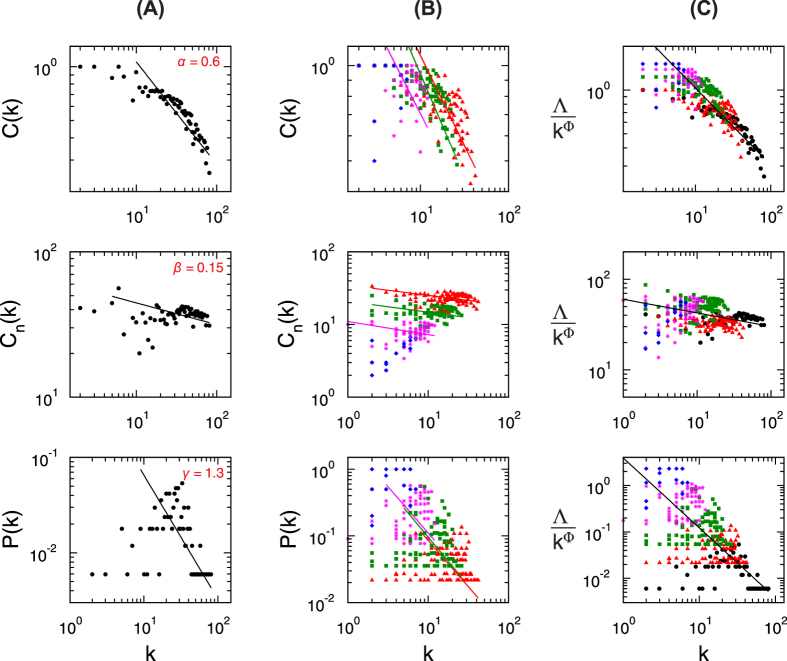
Topological characteristics in the case of the brain network of human species: (**A**) for the whole brain network, (**B**) for modules and sub-modules at various levels, and (**C**) scaled for all modules and sub-modules in all levels. First, second, and third rows of panels show the characteristics of clustering co-efficient, neighborhood connectivity, and the probability of degree distribution of the species, respectively.

The topological properties of hierarchical network, which involve emergence of well-defined modules with few sparsely distributed hubs, can be characterized by three topological parameters, namely, probability of degree distribution *P*(*k*), *P*(*k*) ~ *k*^−*γ*^, with *γ* ≤ 2.0^22^, clustering co-efficient *C*(*k*), *C*(*k*) ~ *k*^−*α*^, with *α* ≈ 1^23^, and neighborhood connectivity *C*_*n*_(*k*), *C*_*n*_(*k*) ~ *k*^−*β*^ with *β* ≈ 0.5^24^; and follow power-law distributions with degree *k*^25^. To prove the existence of power law, we implement the statistical technique proposed by Clauset *et al*.^26^ (Methods) to the data and found that the calculated *p*-values are above the threshold value 0.1 (Table 1), which indicates that power-law distribution could not be ruled out. However, as some of the data set are sparsely distributed, we remove (as required) few low-degree nodes and maximum number of larger degree data are retained during the fitting procedure. The set of the exponents (*γ, α, β*) of the three distributions (*P*(*k*), *C*(*k*), *C*_*n*_(*k*)) calculated using network theory for the four species, namely, *C. elegans*, cat, monkey, and human, are given by: *C. elegans* → (2.0, 0.65, 0.28); cat → (1.8, 0.8, 0.25); monkey → (1.7, 0.73, 0.2); and human → (1.3, 0.6, 0.15), showing hierarchical features in all the four brain networks studied (Figs 1, 2 and 3 panels (A), and Fig. 4 panels (A)). The organization of modules and smaller modules at different *levels* (*level-1* modules are the set of modules constructed from the networks, *level-2* sub-modules are the set of all sub-modules constructed from *level-1* modules, and so on) of the four species (Fig. 1) shows the hierarchical properties. The smallest module in each brain network (*C. elegans*, cat, monkey, and human), from which all the three distributions (*P*(*k*), *C*(*k*), and *C*_*n*_(*k*)) can be calculated, is found to be a triangular motif (Fig. 1). So one can think of triangular motif as the fundamental regulator of each brain network.

### Scaling of modules at different *levels*

We now study the topological properties of modules at different *levels* in each brain network (Fig. 3 panels (A) and (B)). The plotted *P*(*k*) versus *k* of *C. elegans* of larger modules and sub-modules at different *levels* (Fig. 1: *level-1*, C3; *level-2*, SC13; *level-3*, SC13-3) show nearly parallel straight lines of power-law fits of *P*(*k*) with *k* at log-log scale. The data points of the modules and sub-modules at different *levels* along with data of whole network are rescaled to a single plot using one parameter scaling theory (see Methods)^27^,^28^,^29^. Statistical procedure to prove the existence of any power-law distribution^26^ in the scaled data is implemented and the corresponding *p*-value is found to be 0.2876, thus fails to reject the power-law hypothesis. The power-law fitting of *P*(*k*) on the rescaled data gives *γ* = 1.8. The same process is done to calculate clustering co-efficient *C*(*k*) and neighborhood connectivity *C*_*n*_(*k*) of the same data set of modules and sub-modules at different *levels*, rescaled the data set with original whole network using this one parameter scaling method, fitted with power-law equations with *k* in log-log scale and found their exponents to be *α* = 0.7 and *β* = 0.27, respectively.

Similar network analysis is done for cat, monkey, and human brain networks also (Figs 3 and 4). Scaling of the set of data of each species is done using the one parameter scaling method, and exponents of the respective distributions which specify topological characteristics of the networks are determined by fitting the rescaled data with the respective distribution equations (Fig. 3). The set of exponents (*γ, α, β*) of the distributions of the scaled data of the other three species are: cat → (1.7, 0.7, 0.25), monkey → (1.6, 0.68, 0.25), and human → (1.5, 0.53, 0.15), respectively.

### Fractal nature of modules at different *levels*

**Figure 5:**
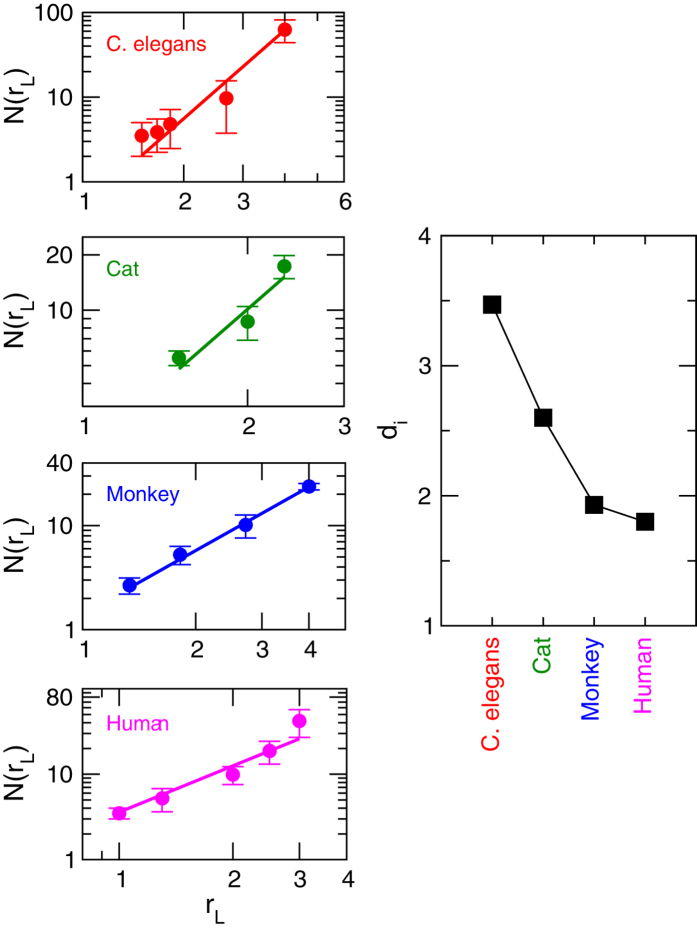
Scaling behavior of modules and sub-modules at various levels of organization of *C. elegans*, cat, monkey, and human species by calculating network mass (number of nodes) as a function of diameter. The right-hand panel shows the respective fractal dimensions of the brain networks of the species.

The characterization of self-similar structures in network can be studied from the evolution of structures (number of nodes in the structures) in the network with path length^14^. We calculated the number of nodes *n* and diameter *R*_*L*_ in each module or sub-module in a certain *level L*, and then average over number of nodes *N* = 〈*n*〉 and path lengths *r*_*L*_ = 〈*R*_*L*_〉 of all modules are taken. The evolution of *N*(*r*_*L*_) as a function of *r*_*L*_ for all *levels* in each network of the four species is shown in Fig. 5. The behavior of *N*(*r*_*L*_) with *r*_*L*_ in all the brain networks follows the following power law:


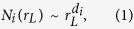


where *d*_*i*_ = {*d*_*i*_; *i* = 1, 2, 3, 4} is the set of Hausdorff fractal dimensions of brain networks of the four species. The value of *d*_*i*_ of brain network of each species can be calculated by fitting the power-law equation (1) on *N*(*r*_*L*_) versus *r*_*L*_ data of the respective species (Fig. 5), and the calculated fractal dimensions of all the four species are shown in Fig. 5. The fractal dimension is found to be the largest for *C. elegans* (*d*_*ce*_ = 3.47) and smallest for human (*d*_*h*_ = 1.8). Since fractal dimension is directly related to surface morphology of any system, larger value of fractal dimension may probably indicate larger disorder in network organization^30^. Its smaller value in the brain network of higher level species may reveal the organization of the network is more ordered and systematically self-organized^31^.

**Figure 6:**
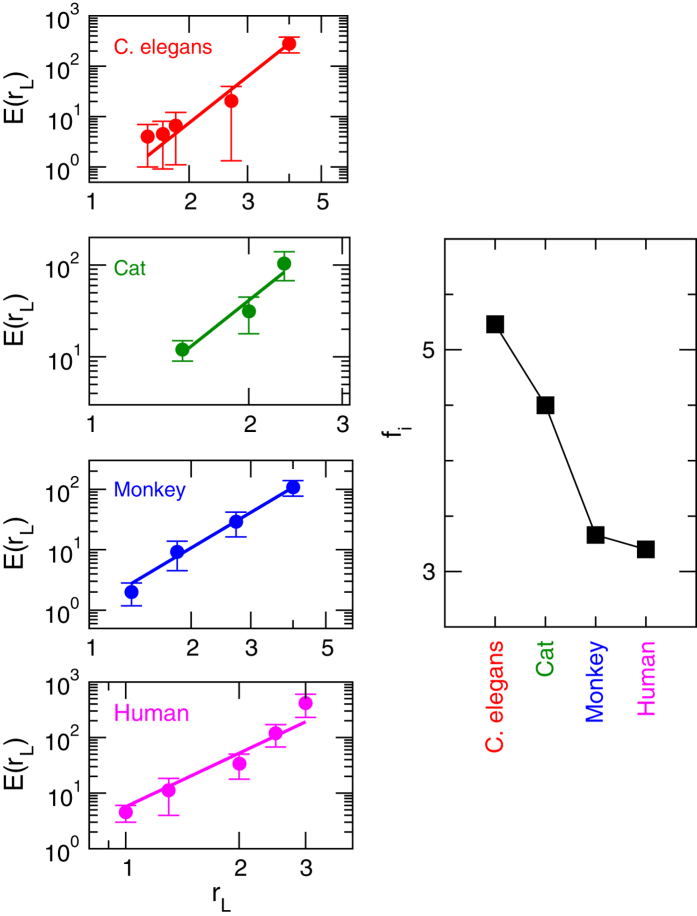
Fractal nature of modules and sub-modules at various levels of organization of *C. elegans*, cat, monkey, and human species by calculating the number of intra-edges as a function of diameter. The right-hand panel shows the variation in the values of fractal dimension of the brain networks of the species.

To understand our claim of fractal nature of organization of modules (relating to the interaction) in brain networks, we now calculate the number of edges (*e*) and diameter (*r*_*L*_) in each module of a certain *level* (for *j*th module of *L*th *level*: 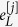, 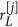), and then obtain average edges and diameter of the modules of the *level L* of *i*th species given by 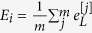 and 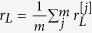, where *m* is the number of modules/sub-modules at level *L*. The behavior of *E*_*i*_ as a function of *r*_*L*_ again obeys the following power law (Fig. 6):


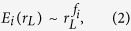


where *f*_*i*_ = {*f*_*i*_; *i* = 1, 2, 3, 4} is the set of fractal dimension relating to edges of modules and sub-modules of brain networks of the four species. The fractal dimension values of the respective species in this case are found to be higher than the respective values of fractal dimension calculated using network mass or network node number, i.e. *f*_*i*_ > *d*_*i*_; however, both *d*_*i*_ and *f*_*i*_ show the similar nature (Figs 5 and 6 right panels).

**Figure 7:**
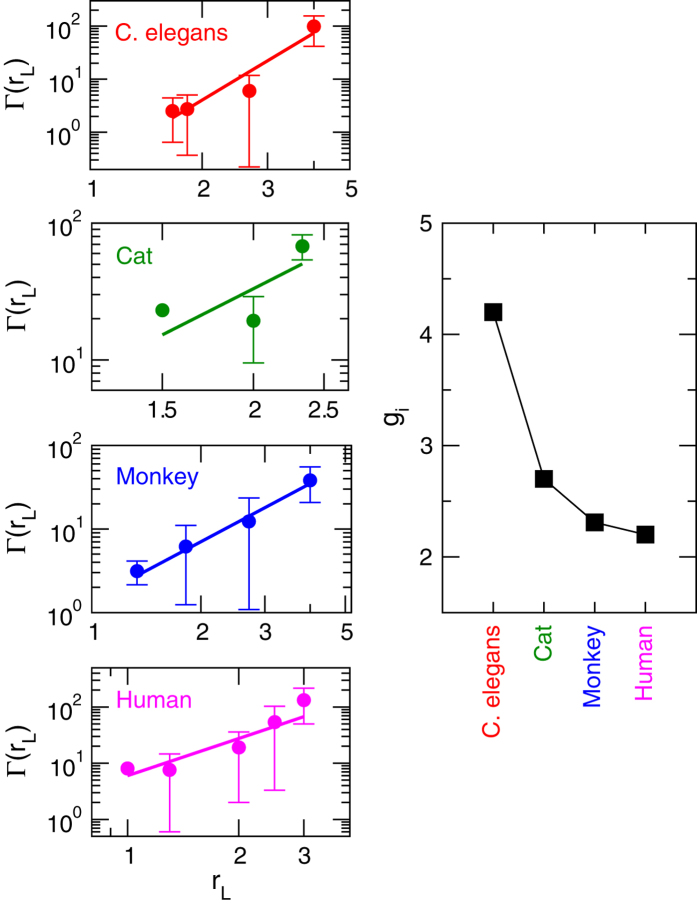
Self-similar properties of modules and sub-modules at various levels of organization of *C. elegans*, cat, monkey, and human species by calculating inter-modular edges of all modules and sub-modules as a function of diameter. The right-hand panel shows the respective fractal dimension values of the brain networks of the species.

The nature of organization of modules among different *levels* can also be investigated by studying the inter-modular interaction among the modules and sub-modules. We calculate the number of edges between any pair of modules in a particular *level L* of brain network of *i*th species, average over all the inter-modular edges of all possible pairs of modules/sub-modules given by Γ_*i*_, and then study the variation of Γ_*i*_ as a function of average diameter of all modules/sub-modules *r*_*L*_ in the level (Fig. 7). The variation of Γ_*i*_ with *r*_*L*_ for all brain networks of the four species *C. elegans*, cat, monkey, and human (Fig. 7) shows power-law behavior (fitted line to the data points) given by


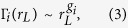


where *g*_*i*_ = {*g*_*i*_; *i* = 1, 2, 3, 4} is the set of fractal dimensions of brain networks of all the four species. This power-law nature reveals the fractal nature of the inter-modular organization of the brain networks. The power-law behavior of mass (number of nodes), intra-modular, and inter-modular edges of modules and sub-modules in all the *levels* of brain networks shows the fractal nature in the organization of brain networks.

The scaling and fractal properties of modules and sub-modules at different *levels* of the brain network of each species probably connect the topological organization of the modules and sub-modules to their functionalities and working relationships among them, within and among the *levels*. Further, self-organization among these modules and sub-modules could facilitate quick communication by minimizing the local and global energy expenditure in communication within the network. The increase in the value of fractal dimensions *d*_*i*_ and *f*_*i*_ given by equations (1) and (2) (Figs 5 and 6) indicates the increase in complexity of the network^13^,^30^. Since the values of *d*_*i*_ and *f*_*i*_ are minimum in human brain network as compare to the other three species, the modules and sub-modules in this network are more ordered and self-organized locally as well as globally as compared to the brain networks of the other three species. This efficient self-organization in the brain network of a certain species might reflect to the fast brain cognition in that species.

### Scaling in centralities and organization

**Figure 8:**
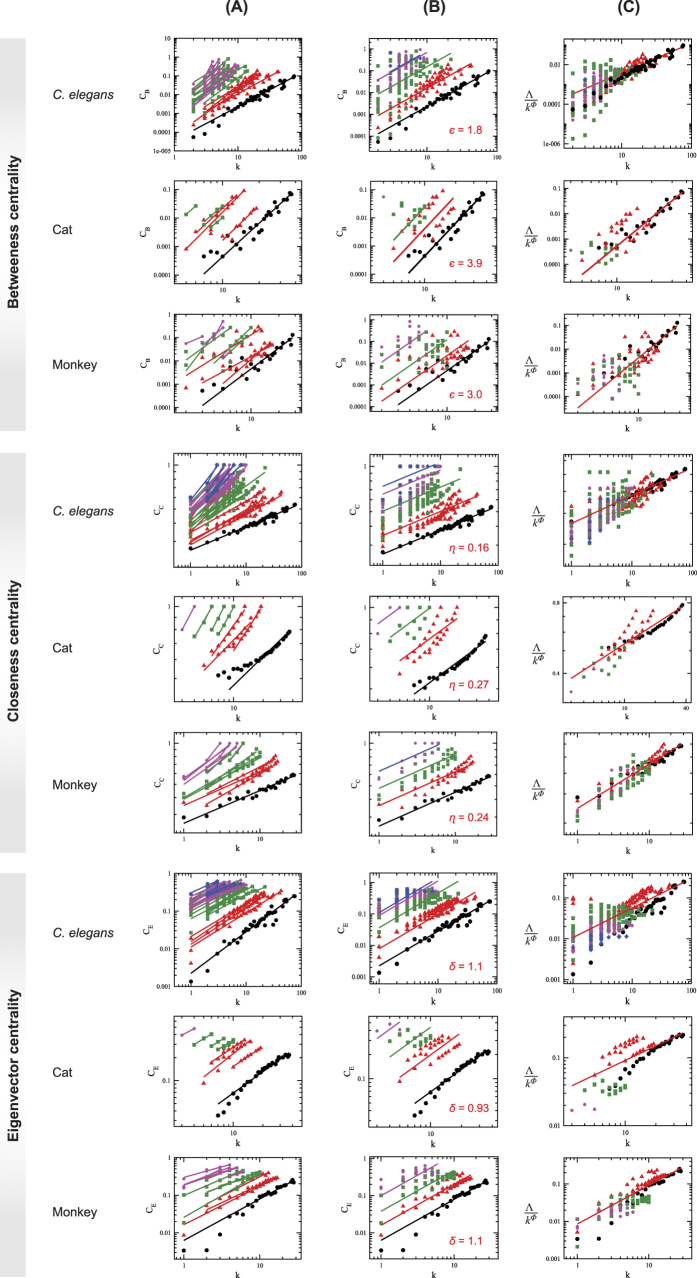
Scaling in centrality parameters of brain networks of *C. elegans*, cat, and monkey species: (**A**) centrality measures of all modules and sub-modules at various levels of the brain networks, (**B**) power-law fits on distribution of the centrality measures of each level, and (**C**) scaled centrality data of all modules and sub-modules into a single curve. The first three upper rows of panels are for betweenness centrality, next three rows are for closeness centrality, and last three rows are for eigenvector centrality of the three species.

**Figure 9:**
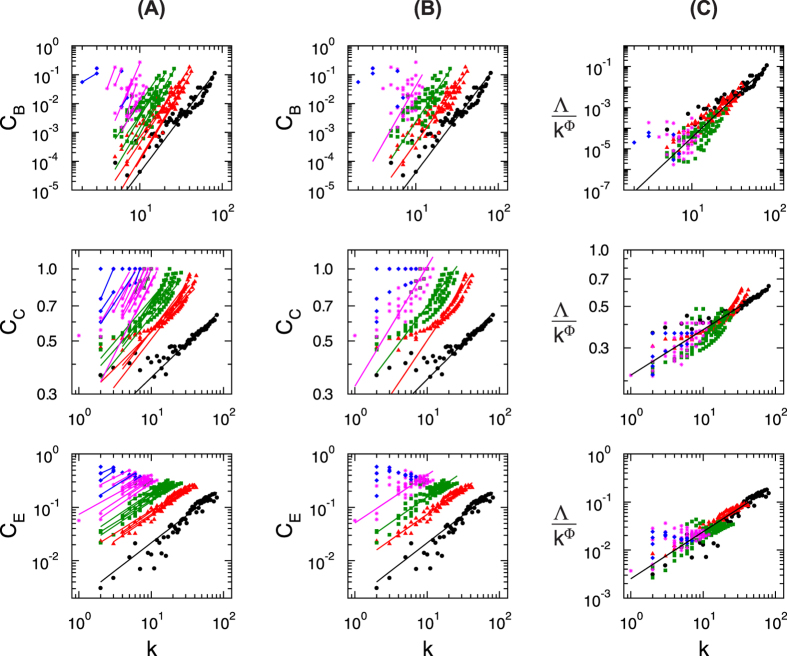
Scaling in centrality parameters of the brain network of human species: (**A**) centrality measures of all modules and sub-modules at various levels of organization, (**B**) power-law fits on distribution of the centrality measures of each level, and (**C**) scaled centrality data of all modules and sub-modules into a single curve. First, second, and third rows of panels represent the characteristics of betweenness centrality, closeness centrality, and eigenvector centrality of the species, respectively.

The betweenness centrality of *C. elegans*, for the whole brain network, modules, and sub-modules at different *levels*, increases as degree of the network increases (Fig. 8 upper panel) which indicates that hubs in the network has significant roles in intra- and inter-modular/sub-modular signal processing at different *levels*. Since high value of betweenness centrality of a node of degree *k* reveals that the node could establish quick communication with other nodes in the network/module/sub-module through short paths^32^,^33^,^34^, hubs in the *C. elegans* brain network may interfere in various network regulations and act as a controller of the network. Removing such few hubs emerged in the hierarchical brain may cause rewiring of the nodes in the modules and sub-modules at various *levels* that may introduce new hierarchical topology of modules/sub-modules. The study of betweenness centrality of modules and sub-modules at different *levels C*_B_ as a function of degree *k* follows a power-law distribution given by


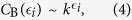


where 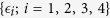 is the set of power-law exponents for different *levels* indicated by *i*. The fitted lines on the data of modules and sub-modules show nearly parallel feature, and the values of 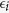 are in the range [1.23, 3.78]. It is also found that *C*_B_ of sub-modules changes significantly with the variation in degree *k* in each *level* (Fig. 8 uppermost panel (A)). The data of all modules/sub-modules in a certain level are fitted with equation (4), and it is found that the fitted lines on the four different *levels* are approximately parallel (Fig. 8 uppermost panel (B)). All the data of modules/sub-modules in all the levels are then scaled using one parameter scaling method (see Methods) and finally fitted with equation (4) (Fig. 8 uppermost panel (C)), and the exponent is found to be 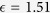. Statistical procedure for testing the existence of power-law behavior^26^ is implemented, and the resulting *p*-value is found to be 0.3384 which is larger than the threshold value (Table 1). Thus, the scaled data set follows power-law nature. We also observe that smaller modules have better communication among the nodes within each module^35^. Further, as the smaller modules reach the motif level (here triangular motif), each node in the motif has equal importance (similar due to same degree in each node), with single value of *C*_B_. If a particular module at any *level* is considered, *C*_*B*_ increases as *k*, indicating significant important role of hubs in information processing within the module^36^,^37^,^38^,^39^,^40^. The scaling of *C*_*B*_ data for all modules and sub-modules in a particular network follows one parameter scaling law (single power-law-fitted line on the scaled data as shown in Figs 8 and 9 panels C) which reveals similar topological constitution of the network at all modules and sub-modules at various levels of organization of the network. We follow the same process of analysis to cat, monkey, and human brain network data also, and found similar behavior in *C*_B_ as a function of *k* given by the scaling law of equation (4) (Fig. 8, second and third rows; and Fig. 9, first row), and their exponents are found to be 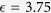, 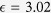, and 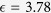, respectively. The scaling in the power-law behavior of *C*_B_ indicates fractal behavior of the modules/sub-modules at various levels up to the motif level.

Closeness centrality (*C*_C_) is another measure of centrality which describes how quickly an information from (by) a node can be propagated (received) to (from) the rest of the network, and can be characterized by the inverse of average distance between a given node with other nodes in the network^41^. The calculated *C*_C_ of *C. elegans* as a function of degree *k* increases as *k* increases (Fig. 8, fourth row panel (A)) which indicates that the increase in *C*_C_ with *k* exhibits shorter average path length (see equation (9) in Methods) meaning faster information processing of the node with the rest of the brain network. This means that larger hubs (larger *k*) are able to communicate with the rest of the nodes in the brain network of *C. elegans* faster than the nodes with smaller *k*, which is true for hubs in modules/sub-modules other than motif where every constituting nodes have same *k*. The data of modules and sub-modules at various levels obey the following power-law behavior:


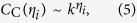


where the set {*η*_*i*_; *i* = 1, 2, 3, 4} are closeness centrality exponents at various levels. The fitted curves with equation (5) on the data of modules and sub-modules at various levels of the network are approximately parallel (Fig. 8, fourth row panel (A)). The data of all modules/sub-modules at each level are fitted with equation (5) (Fig. 8, fourth row panel (B)), and it is found that the fitted lines are approximately parallel. The data from all levels are then scaled using one parameter scaling method to a single curve and the scaled data are fitted to a power law given by equation (5) (Fig. 8, fourth row panel (B)), and the exponent is found as *η* = 0.16; the existence of the power-law distribution has been statistically confirmed using techniques of Clauset *et al*.^26^ (see Table 1 and Methods). The same procedure has been used to analyze the data of brain networks of cat, monkey, and human species. The similar behavior in terms of scaling and structural properties are found in the three species, and their brain data follow the power-law behavior given by equation (5) with power-law exponent *η* = 0.28, *η* = 0.26, and *η* = 0.24, respectively. The results show the fractal behavior of closeness centrality which may connect the network topology of brain networks to brain functionality.

Eigenvector centrality (EC) is in favor of highly correlated nodes (which are usually high degree nodes) with rest of nodes in a network, and specific nodes which connect central nodes within the network relating to global network pattern^42^. EC is characterized by well-connectedness in a network^43^, a smooth enough function^44^, and is a good measure of spreading (receiving) power of information of nodes in (from) the network^45^. The calculated EC of the brain network of *C. elegans* (*C*_E_) for the network, modules, and sub-modules at various levels (see Methods) show increase in its values as degree *k* increases, obeying the following power law (for statistical techniques implemented in testing the existence of power-law distribution, see Methods; Table 1),


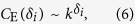


where {*δ*_*i*_; *i* = 1, 2, 3, 4} is the set of EC exponents at various levels. As found in betweenness and closeness centralities, the fitted lines on the data of *C. elegans* brain network, its modules, and sub-modules are nearly parallel with EC exponents in the range [0.5, 1.1]. Similarly, it is also found that as one goes towards higher levels, i.e. smaller module levels, *C*_C_ also increases comparatively. All the data of modules/sub-modules at each level when fitted with equation (6) are found to be approximately parallel (Fig. 8, seventh row panel (B)). Data from all levels are then scaled in a single data set and fitted with equation (6) (Fig. 8, seventh row panel (C)); the exponent is found as *δ* ~ 0.72 (for statistical testing of power-law distribution, see Methods; Table 1).

Similar behavior is found in the brain networks of cat (Fig. 8, eighth row panels (A), (B), and (C)), monkey (Fig. 8, ninth row panels (A), (B), and (C)), and human (Fig. 9, third row panels (A), (B), and (C)) following the same scaling power law given by equation (6), and found the values of *δ* to be 0.71, 1.03, and 0.98, respectively.

### Evidence of self-organization: local-community-paradigm (LCP) approach

**Figure 10:**
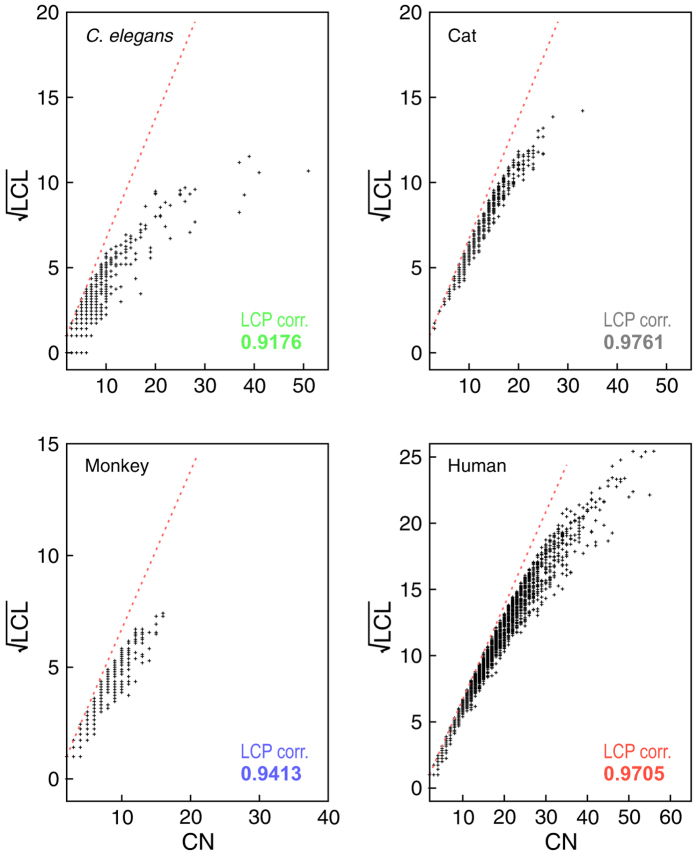
LCP-DP plots of brain networks of the four species, namely, *C. elegans*, cat, monkey, and human. The LCP-corr are also calculated for each network (given in each figure panels).

**Figure 11:**
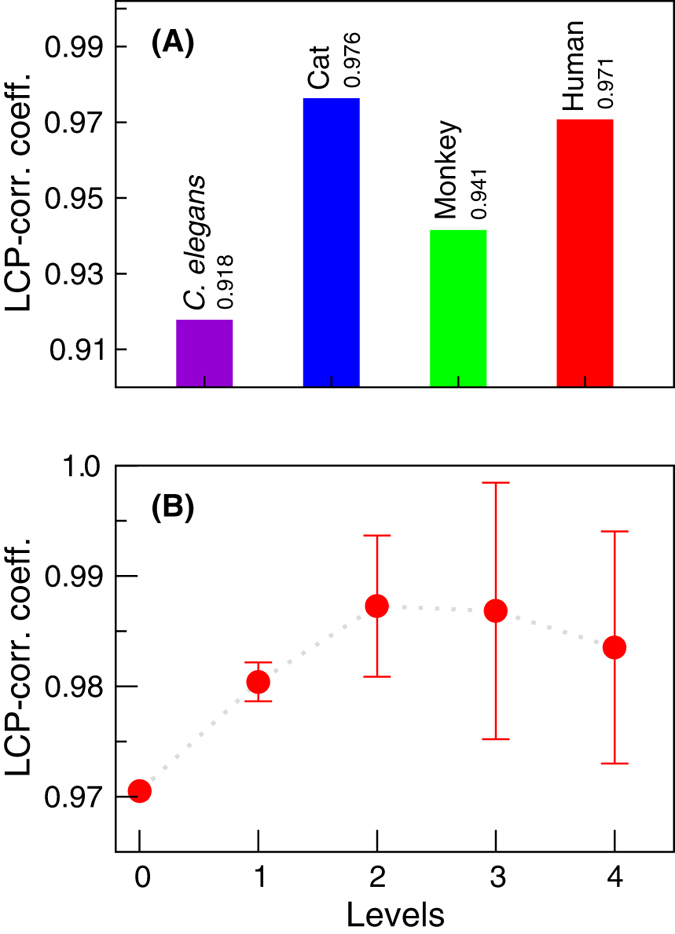
(**A**) LCP-corr calculated for brain networks of the four species, *C. elegans*, cat, monkey, and human. (**B**) Variation in the calculated average LCP-corr for human brain network as a function of network level.

The LCP-decomposition-plot (LCP-DP; see Methods) for all four complete networks (*C. elegans*, cat, monkey, and human) are shown in Fig. 10, and the range of local-community sizes of these four networks are [1–52] (*C. elegans*), [1–34] (cat), [1–18] (monkey), and [1–57] (human). The results show that modules of *C. elegans* are more sparsely distributed as compared to others, but modules in human network are more compact. The calculated LCP-correlation (LCP-corr) values for all these species are large (>0.9; Fig. 11 upper panel) indicating strong LCP networks that are dynamics and heterogeneous, which facilitate network evolution and reorganization^21^. This indicates that these networks are organized with diverse modules/sub-modules at various levels of organization in hierarchical manner, which prohibits a central control in these networks with hub/hubs manipulations in these networks. The removal of hub/hubs from these networks do not cause breakdown of these networks due to strong self-organization in these networks.

**Figure 12:**
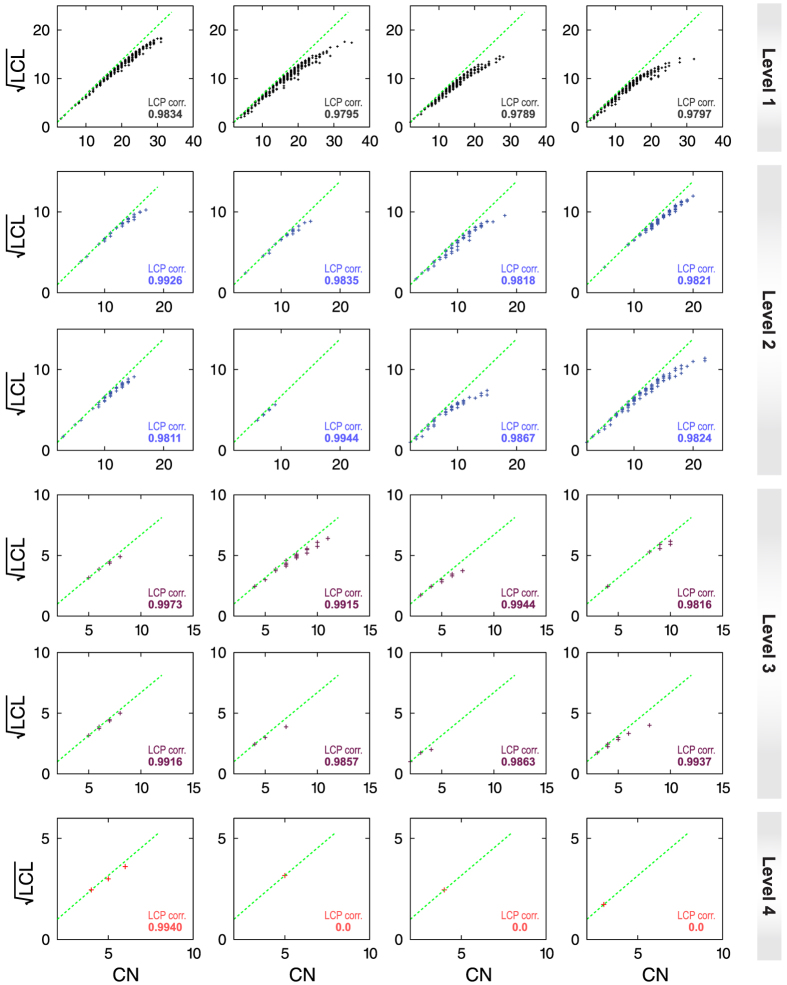
LCP-DP plots of modules/sub-modules at various levels of organization in human brain network. The LCP-corr are also calculated for each module/sub-module at various levels (given in each figure panels).

We then analyzed the human brain network to check the maintenance of this self-organization at various levels of network organization (Fig. 12) using LCP technique. The human brain network that we studied is organized in four different levels of organization (Fig. 12). The results show that even though the sizes of modules/sub-modules at different levels of organization are different (maximum *local-community* size is 35) and decreases as level of organization increases where some of the sub-modules at *level-4* have single overlapped points, the points in the LCP-DP are not scattered showing compact constitution of the nodes in each module/sub-modules. We then calculated LCP-corr of all the modules/sub-modules at various levels (Fig. 12). The average values of LCP-corr at each level (modules having zero LCP-corr are not taken in average) are larger than the LCP-corr value of complete network and do not change within the error bar (Fig. 11 lower panel). This indicates the maintenance of self-organization of modules/sub-modules with better compactness and efficient information processing. The fractal and scaling nature of the brain networks could be as a consequence of self-organized behavior of these brain networks at various levels of organization.

## Discussion

The findings of our study suggest that the fundamental working principle of brain (in both lower and higher level species) is a system level topological self-organization. The fractal nature and scaling properties of these brain networks show self-similar organization of various topographical modules/sub-modules at every levels of constitution, which may relate to the functional brain organization, and energy cost in information transfer within and among the levels of organization is minimized. In addition, the few sparsely distributed hubs are tightly bound in their respective module and interfere functionalities of their own module, but could not influence rest of the modules at various levels in brain networks. In terms of inter-modular and intra-modular interaction edges, each brain network still show fractal nature which indicates systematic self-similar information processing at every levels and their interference. The decrease in the values of fractal dimension in going from *C. elegans* to human (lower to higher species) shows that the organization of brain networks (in terms of signal processing, topological characteristics, and modular organization) is more ordered and self-organized systematically in higher species. Such topological properties in brain networks allow efficient information processing, constitution of fractal laws in the organization, and controlled behavior of hubs in the global network properties.

The centrality measures (betweenness, closeness, and eigenvector centralities) of brain networks, its modules, and sub-modules show increase in their values with degree showing that hubs behave as most influencing nodes in the modules/sub-modules they are embedded. These hubs act as central in the local module/sub-module, and they become local quick information spreader and receiver in the network. However, removing one hub in such situation does not cause the network breakdown because of the system level organization of the network through modules and smaller modules which are compact with their own fundamental rules. The centrality data of the brain network, modules, and sub-modules at different levels can be scaled into a single power-law behavior showing fractal nature. This exhibited fractal nature in the brain network could be the consequence of the emergence of a few most influencing hubs in each module/sub-module at any level of the network except at the level of motif where all the nodes in it have equal degree. Therefore, in the brain network, modules, and smaller modules, most popular node/nodes always exist and they take maximum responsibility in regulating the network/module/sub-module. However, these hubs’ interference in the network is controlled (due to limited number of links to the modules/sub-modules) in such a way that they cannot control the other modules/sub-modules but can regulate them.

The scaling properties in brain networks reveal complicated self-organization of the network at various topological levels, and it could probably explain systematic organization of functional modules via weak interaction among them. This topographic organization may induce the origin of brain functionalities even at the absence of few hubs or modules. However, the properties of this static network do not fully explain the working principle of the complicated brain network, its dynamics, and functional relationships. The studies on dynamics and multi-scaled network approach may highlight further interesting insights on brain organization/reorganization.

## Methods

### Data sources

In this paper, the connection matrices of (1) *C. elegans* neuronal system, (2) 52 cortical areas in cat species, (3) 71 cortical areas in Macaque monkey species, and (4) 168 brain regions in human species are studied.

The *C. elegans* neuronal connectivity data set is adapted from Achacoso & Yamamoto^46^, the compilation of which is based on the work of White *et al*.^47^ in which the neuronal connection were traced with electron microscope reconstructions. Further modifications are the removal of 20 neurons in the pharyngeal nervous system which have no internal connection information^46^ and the additional removal of three other neurons (AIBL, AIYL, and SMDVL) considering their lack of spatial information^48^,^49^. Finally, 277 neurons sharing 2102 synaptic connections are considered for further topological analysis (data set available at http://www.biological-networks.org).

The cat connection matrix used in this study is derived from the original article by Scannell *et al*.^50^. In their paper, they collected information on the thalamo-cortico-cortical connections from many published studies, and applied the methods of non-metric multi-dimensional scaling, optimal set analysis, and non-parametric cluster analysis to derive the connection matrix of the 53 cortical areas and 42 thalamic nuclei. Their connection matrix is relatively weighted (0, 1, 2, and 3) according to the connection strength (absent/unreported, weak, intermediate, and strong, respectively) between each region. In this paper, only those connections among the 52 cortico-cortical areas are studied (after “Hipp” area is omitted)^51^. The relative weighting is discarded and only the presence or absence of connection is considered in the respective adjacency matrix. The resulting final matrix has 52 cortical areas and 820 cortico-cortical connections.

Collecting information from the neuroanatomical studies, Young^52^ applied the method of optimization analysis to map the cortico-cortical connections between 73 cortical areas of interest in the entire cerebral cortex of Macaque monkey. The connection matrix of Macaque monkey used in this paper is also based on the study of Young^52^, with a modification as mentioned in Sporns & Zwi^51^ in which two areas of interest (Hipp and Amyg) are removed resulting to a total of 71 cortical areas with 746 interconnections (data set available at https://sites.google.com/site/bctnet/datasets).

Finally, for the human brain, diffusion-weighted imaging (DWI) data for 89 subjects (age = 11–77 years) were obtained from the publicly available Enhanced Nathan Klein Institute Rockland Sample (NKI)^53^. All images were acquired using 3.0 T Siemens Magnetom TrioTim scanner. A MPRAGE sequence (TR = 1900 ms; TE = 2.52 ms; voxel size = 1 mm isotropic) was used to obtain T1-weighted images. A high spatial and angular resolution (TR = 2400 ms; TE = 85 ms; voxel size = 2 mm isotropic; *b* = 1500 s/mm^2^; 137 gradient directions) was used to collect DWI data. Detailed pre-processing steps for DWI data are given elsewhere^54^. Steps include: conversion of DWI data to 4D volumes, cleaning the data of motion and other artifacts using DTIPrep^55^, structural unwarping of the cleaned 4D diffusion volumes followed by linear registration of the average *B*_0_ to the T1 volume in stereotaxic space. Processing of the 4D diffusion volumes was done with FSL’s *bedpostx*. Probabilistic tractography was performed with FSL’s *probtrackx* package^56^ for the Cambridge brain parcellation^57^ resulting to connectivity matrices for each subject which were then averaged across the group to obtain a single 168 × 168 DWI connectivity matrix.

### Graph construction and network parameters

The connection matrices (adjacency matrices) from the above-mentioned data sets are used to generate undirected graphs by using *igraph* R package^58^. For identifying communities in these graphs, the leading eigenvector spectral graph partitioning method (for which algorithm is available in *igraph* package) is implemented^59^. In this method, the modularity term is expressed in terms of eigenvalues and eigenvectors of a modularity matrix, and the partitioning is done using multiple leading eigenvectors that optimizes the modularity^60^. The communities are then grouped into each topological level. For each graph/subgraph we use the NetworkAnalyzer^61^,^62^ and CytoNCA^63^ plug-ins in *Cytoscape* for finding required network parameters such as degree, clustering coefficient, neighborhood connectivity, betweenness centrality, closeness centrality, and eigenvector centrality.

#### Degree distribution

The degree represents a centrality measure that indicates the number of communications a node maintains with other nodes in a graph. The degree distribution (*P*(*k*)) which is the probability that a randomly chosen node has a degree *k* represents an important parameter that helps us to identify whether a graph is random, scale free, hierarchical, etc.


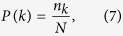


where *n*_*k*_ represents the number of nodes with degree *k* and *N* is the total number of nodes in the graph.

#### Neighborhood connectivity

Neighborhood connectivity of a node *i* represents the average connectivities (average degrees) of the nearest neighbors of node *i*^64^.

#### Clustering co-efficient

Clustering co-efficient is a measure of how strongly a node’s neighborhoods are interconnected. Graph theoretically clustering coefficient is the ratio of the number of triangular motifs a node has with its nearest neighbor to the maximum possible number of such motifs. For an undirected graph, clustering coefficient (*C*_*i*_) of the *i*th node can mathematically be expressed as


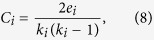


where *e*_*i*_ is the number of connected pairs of nearest-neighbor of the *i*th node, and *k*_*i*_ is the degree of the *i*th node.

#### Centrality measurement

In addition, other important centrality measures include (1) closeness centrality, (2) betweenness centrality, and (3) eigenvector centrality. Centrality measures are helpful in identifying influential node(s) in a graph.

#### Closeness centrality

Closeness centrality (*C*_C_) of a node is the reciprocal of the mean geodesic distance between the node and all other nodes reachable from it^41^. Therefore, it represents how fast information is spread from the node to other nodes in the network. Thus, for a node *i*,


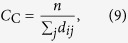


where *d*_*ij*_ represents the geodesic path length from nodes *i* to *j*, and *n* is the total number of vertices in the graph reachable from node *i*.

#### Betweenness centrality

Betweenness centrality of a node is the measure of the extent to which the node has control over the communication of other nodes. Betweenness centrality (*C*_B_) of a node *v* is computed as follows^65^,^66^,^67^:


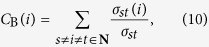


where **N** is the set of nodes, *s* and *t* are nodes in the graph different from *i, σ*_*st*_ is the number of shortest path from *s* to *t*, and path through *i* in the case of *σ*_*st*_(*i*). The betweenness centrality value is normalized by dividing with the number of node pairs (excluding node *i*).

#### Eigenvector centrality

Eigenvector centrality of a node *i* (*v*_*i*_) in a network is proportional to the sum of *i*’s neighbor centralities^68^, and it is given by


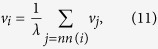


where *nn*(*i*) indicates nearest neighbors of node *i* in the network. *λ* is the eigenvalue of the eigenvector *v*_*i*_ given by


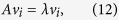


where *A* is the adjacency matrix of the network. The principal eigenvector of matrix *A*, which corresponds to maximum eigenvalue *λ*_max_, is taken to have positive eigenvector centrality scores^45^.

#### Modularity

Finally, modularity is the measure of how well a network is divided in communities^69^. Modularity (*Q*) is express as follows:


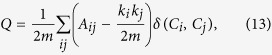


where *m* is the total number of edges in the community, *A*_*ij*_ is the adjacency matrix of size *i* × *j, k* represents degrees, and the *δ* function yields 1 if nodes *i* and *j* are in the same community.

#### Statistical testing for power-law distribution

The statistical technique we used in our study to conclude the existence of a power-law distribution in an empirical data revolves around the method suggested by Clauset *et al*.^26^. The method has been used as an addition to the older least-squares linear regression technique. Clauset *et al*. techniques mainly involve two steps. First, estimating the lower bound (*x*_min_) and exponent (*α*) of a fitted model. Second, perform the goodness-of-fit test of the empirical data against the fitted model.

In this technique, *x*_min_ is selected so as to minimize the Kolmogorov–Smirnov (KS) statics which can be defined as


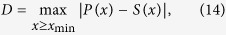


where *P*(*x*) is the cumulative distribution function (CDF) of the best-fit power-law model considering the data region *x* ≥ *x*_min_ and *S*(*x*) represents the empirical CDF.

The exponent *α* is then derived using the maximum likelihood method, and for the continuous empirical data the maximum likelihood estimator (MLE) can be expresses as


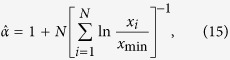


where {*x*_*i*_: *i* = 1, …, *N*} represents the set of data points for which *x* ≥ *x*_min_.

For the case discrete data points, MLE 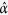 is the solution of the equation


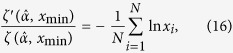


where *ζ*(*α, x*) is the generalized zeta function, and *ζ*′ denotes the differentiation with respect to the first argument^26^.

Second, the theoretical power-law model that is generated from the empirical data are then subjected to KS goodness-of-fit test. For this, many synthetic data sets are generated on the basis of the fitted theoretical power-law parameters. The distance is then calculated between CDFs of the empirical and modeled data (say *D*_m_), as well as between the synthetic and model data (say *D*_s_). Finally, *p*-value is calculated considering the fraction of synthetic data for which *D*_s_ > *D*_m_.

The existence of a power-law distribution in an empirical data could not be rejected if *p*-value is larger than 0.1. For example, if a total number of 2500 synthetic data samples are considered, the claim for the existence of a power-law distribution is plausible only when less than 250 samples satisfy *D*_s_ > *D*_m_ relation. We use the “poweRlaw” package^70^ and “plfit.r” script in R for our analysis (see also http://tuvalu.santafe.edu/aaronc/powerlaws/).

#### Scaling nature of topological parameters

The data of topological parameters (probability of degree distribution, clustering co-efficient, and neighborhood connectivity) and centrality parameters (betweenness, closeness, and eigenvector centralities) of the network, modules, and sub-modules at various levels (Figs 3 and 8) in log-log plot show approximately parallel power-law fit lines. We follow one parameter scaling theory^27^,^28^,^29^ to scale the data given by


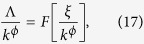


where *F* is a scaling function. For topological parameters Λ(*k*) = *P*(*k*), *C*(*k*), *c*_*n*_(*k*) and for centralities Λ(*k*) = *C*_B_(*k*), *C*_C_(*k*), *C*_E_(*k*) with corresponding *ϕ* values after fit. The calculated *ξ* after fitting each data of network/module/sub-module corresponds to the minimum path length of the network/module/sub-module approximately. This fitting procedure gives us *F* ≈ *constant*. Hence, we found the following scaling law:


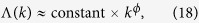


where *ϕ* = {−*α*, −*β*, −*γ*} for *C*(*k*), *C*_*n*_(*k*), and *P*(*k*), respectively, and 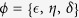 for *C*_B_(*k*), *C*_C_(*k*), and *C*_E_(*k*), respectively.

#### Local-community-paradigm (LCP) approach

The LCP-decomposition-plot (LCP-DP) is one way of representation of topological properties of a network in two-dimensional parameter space of common neighbors (CN) index of interacting nodes and local community links (LCL) of each pair of interacting nodes in the network, and it provides information on number, size, and compactness of communities in a network, which can further be used as a measure of self-organization in the network^21^. The CN index between two nodes *x* and *y* can be calculated from the measure of overlapping between their sets of first-node-neighbors *S*(*x*) and *S*(*y*) given by, 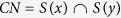. The possible likelihood of interaction of these two nodes could happen if there is significant amount of overlapping between the sets *S*(*x*) and *S*(*y*) (large value of CN), and therefore increase in CN reflects the increase in compactness in the network, which could indicate faster information processing in the network. Further, the LCLs between the two nodes *x* and *y*, whose upper bound is defined by, 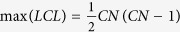, is the number of internal links in local-community (LC), which is strongly inter-linked group of nodes. Then, these two nodes most probably link together if CN of these two nodes are members of LC^21^. LCP-DP has been studied on many types of networks and found to have a linear dependence between CN and 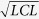. The LCs calculated using LCP approach approximately correspond to the modules/sub-modules at different levels in the network.

The LCP correlation (LCP-corr) is the Pearson correlation co-efficient of CN and LCL defined by 
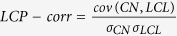
 with *CN* > 1, where *cov*(*CN, LCL*) is the covariance between CN and LCL, *σ*_*CN*_ and *σ*_*LCL*_ are standard deviations of CN and LCL, respectively.

## Additional Information

**How to cite this article**: Singh, S. S. *et al*. Scaling in topological properties of brain networks. *Sci. Rep.* **6**, 24926; doi: 10.1038/srep24926 (2016).

## Figures and Tables

**Table 1:** Test results for power-law distribution*.

	Parameters	Species	*n*	*p*-value	Goodness of fits
Whole network	Clustering coefficient	*C. elegans*	39	0.766	0.1114992
		Cat	22	~0.11	0.2635055
		Monkey	20	0.1344	0.1564396
		Human	59	0.12	0.238144
	Neighborhood connectivity	*C. elegans*	36	0.8328	0.1372943
		Cat	25	0.62	0.1514593
		Monkey	24	0.3804	0.2047339
		Human	59	0.6968	0.07790939
	Degree distribution	*C. elegans*	277	0.5568	0.04458331
		Cat	51	0.1132	0.1160309
		Monkey	71	0.1084	0.1052469
		Human	168	0.168	0.07514285
Data set after scaling from all levels	Clustering coefficient	*C. elegans*	318	0.1752	0.1017557
		Cat	70	0.6064	0.08411472
		Monkey	113	0.4284	0.07989214
		Human	308	0.7112	0.04166865
	Neighborhood connectivity	*C. elegans*	351	0.2192	0.05529387
		Cat	70	0.32	0.08908077
		Monkey	124	0.1976	0.1044134
		Human	310	0.8816	0.04186619
	Degree distribution	*C. elegans*	361	0.2876	0.06655764
		Cat	69	0.115	0.1035658
		Monkey	124	0.6324	0.08231645
		Human	310	0.5048	0.05792419
	Betweenness centrality	*C. elegans*	264	0.3384	0.0652874
		Cat	61	0.1148	0.1140632
		Monkey	98	0.3816	0.07636612
		Human	254	0.2776	0.07253436
	Closeness centrality	*C. elegans*	364	0.1728	0.1119073
		Cat	71	~0.1	0.172004
		Monkey	107	0.2084	0.1183037
		Human	280	0.1384	0.08605586
	Eigenvector centrality	*C. elegans*	340	0.608	0.08864998
		Cat	71	0.26	0.1180827
		Monkey	123	0.5508	0.1134687
		Human	299	0.1708	0.08715249

^*^Values calculated against 2500 random sampling.
